# Short‐term heat stress alters redox balance in porcine skeletal muscle

**DOI:** 10.14814/phy2.13267

**Published:** 2017-04-28

**Authors:** Olga Volodina, Shanthi Ganesan, Sarah C. Pearce, Nicholas K. Gabler, Lance H. Baumgard, Robert P. Rhoads, Joshua T. Selsby

**Affiliations:** ^1^ Department of Animal ScienceIowa State UniversityAmesIowa; ^2^ Department of Animal and Poultry SciencesVirginia TechBlacksburgVirginia

**Keywords:** Heat stroke, hyperthermia, mitochondria, oxidative stress, pig

## Abstract

Heat stress contributes to higher morbidity and mortality in humans and animals and is an agricultural economic challenge because it reduces livestock productivity. Redox balance and associated mitochondrial responses appear to play a central role in heat stress‐induced skeletal muscle pathology. We have previously reported increased oxidative stress and mitochondrial content in oxidative muscle following 12 h of heat stress. The purposes of this investigation were to characterize heat stress‐induced oxidative stress and changes in mitochondrial content and biogenic signaling in oxidative skeletal muscle. Crossbred gilts were randomly assigned to either thermal neutral (21°C; *n* = 8, control group) or heat stress (37°C) conditions for 2 h (*n* = 8), 4 h (*n* = 8), or 6 h (*n* = 8). At the end, their respective environmental exposure, the red portion of the semitendinosus muscle (STR) was harvested. Heat stress increased concentration of malondialdehyde (MDA) following 2 and 4 h compared to thermal neutral and 6 h, which was similar to thermal neutral, and decreased linearly with time. Protein carbonyl content was not influenced by environment. Catalase activity was increased following 4 h of heat stress and superoxide dismutase activity was decreased following 6 h of heat stress compared to thermal neutral conditions. Heat stress‐mediated changes in antioxidant activity were independent of altered protein abundance or transcript expression. Mitochondrial content and mitochondrial biogenic signaling were similar between groups. These data demonstrate that heat stress caused a transient increase in oxidative stress that was countered by a compensatory change in catalase activity. These findings contribute to our growing understanding of the chronology of heat stress‐induced intracellular dysfunctions in skeletal muscle.

## Introduction

Heat stress results from an inability to maintain a balance between heat production and loss and is largely dependent on environmental conditions. Heat stress is a significant risk factor of cardiovascular, hemodynamic, and metabolic pathologies (Crandall and Gonzalez‐Alonso 2010; Kones 2011; Pearson et al. 2011; Forslund et al. 2013; Rhoads et al. 2013). Furthermore, heat stress can lead to mortalities in developed and developing nations (Coumou et al. 2013; Azhar et al. 2014; Ghumman and Horney, 2016), as well as increased costs associated with medical care (Schmeltz et al. 2016). In addition to detrimental effects on human health, heat stress results in agricultural losses of approximately $2.4 billion annually due to losses in production (Nigel Key and Marquardt 2014) and costs associated with health care and maintenance of animal welfare (St‐Pierre et al., 2003). Hence, prolonged exposure to environmental hyperthermia poses a multifaceted threat that negatively impacts humans and animals and necessitates the development of etiological interventions.

While there is an obvious negative effect of heat stress at the organismal level, cellular‐ and system‐level modifications induced by heat are poorly understood. Gaining this understanding is complicated by the duration and intensity of hyperthermic exposure as in muscle short‐term exposure of ~30 min (Naito et al. 2000; Goto et al. 2003; Selsby and Dodd 2005; Selsby et al. 2007) appears to be physiologically distinct from long‐term exposure (>2 h) (Ganesan et al. 2016), which are themselves, distinct from intermediate durations (Welc et al. 2013). Thus, it is clear that a temporal pattern exists and it determines whether the heat load is therapeutic or pathologic.

In skeletal muscle, prolonged heat stress impaired muscle growth and caused changes in muscle metabolism, redox balance, and inflammatory signaling in pigs (Montilla et al. 2014; Cruzen et al. 2015; Ganesan et al. 2016). In particular, heat stress‐mediated free radical production in avian skeletal muscle is associated with disruption of complexes I (Huang et al. 2015), II (Mujahid et al. 2009; Kikusato et al. 2010) and III (Huang et al. 2015) of the electron transport chain (ETC). Thus, disruption of mitochondrial metabolism and respiratory function establishes a link between oxidative stress and heat stress‐induced damage. This makes mitochondrial signaling and antioxidant pathways an important subject of investigation. We have previously found increased oxidative stress and inflammatory signaling in oxidative (Montilla et al. 2014; Ganesan et al. 2016), but not glycolytic, skeletal muscle in pigs heat stressed for 12 or 24 h. This may further implicate the mitochondria as key factors underlying cellular pathology associated with heat stress. The aim of the current investigation was to determine the extent to which short‐term heat stress induced oxidative stress and impacted mitochondrial content and biogenesis markers in oxidative skeletal muscle. We hypothesized that 2, 4, and 6 h of heat stress would result in oxidative stress and alter mitochondrial dynamics in oxidative skeletal muscle compared to skeletal muscle maintained under thermal neutral conditions.

## Materials and Methods

### Animals and study design

All procedures were reviewed and approved by the Iowa State University Institutional Animal Care and Use Committee. A detailed description of methods has been previously published (Pearce et al. 2014). Briefly, 32 gilts (63.8 ± 2.9 kg BW) were randomly assigned to 1 of 4 environmental treatment groups matched by body weight: (1) thermal neutral conditions (21°C; ~70% humidity) (*n* = 8) or (2) exposed to heat stress conditions (37°C; ~40% humidity) for 2 h (*n* = 8), (3) 4 h (*n* = 8), or (4) 6 h (*n* = 8). All pigs were given ad libitum access to feed and water. Room temperature and humidity were monitored continuously and recorded every 5 min by a data recorder (Lascar^®^ EL‐USB‐2‐LCD, Erie, PA); rectal temperature, respiratory rate, and feed intake were recorded every 2 h and have been reported previously (Pearce et al. 2014). Upon completing the experiment, pigs were weighed and euthanized by barbiturate overdose followed by exsanguination. The red portion of the semitendinosus muscle (STR) was harvested immediately, frozen in liquid nitrogen, and stored at −80°C.

### Protein extraction

Frozen STR samples were powdered on dry ice and then 50 mg of powder was homogenized in 500 *μ*l of protein extraction buffer (10 mmol/L sodium phosphate and 2% SDS, pH 7.0) and centrifuged at 1500*g* for 10 min at 4°C. Nuclear and cytoplasmic protein was extracted using Nuclear and Cytoplasmic Extraction Reagents Kit (Thermo Fisher Scientific, Inc., Waltham, MA) from 30 mg of muscle powder. Supernatants containing the whole homogenate, nuclear fraction, and cytoplasmic fraction were stored at −80°C until further analysis. Protein concentration in whole homogenate, nuclear fraction, and cytoplasmic fraction was determined using the Pierce BCA Protein Assay Kit (Thermo Fisher Scientific, Inc.). Whole homogenate and the nuclear fraction were used for western blotting to assess relative protein abundance. Enzymatic activities were measured in the cytoplasmic fraction.

### Western blotting

Immunoblotting was performed according to standard techniques. Briefly, protein was diluted to 4 mg/mL in Laemmli buffer. Forty micrograms of protein was electrophoresed on 4–20% PAGEr Gold Precast Gels (Lonza, Walkersville, MD) at 120 V by SDS‐PAGE and transferred onto nitrocellulose membranes (Bio‐Rad, Hercules, CA) at 100 V for 1 h at 4°C. To ensure equal loading, membranes were stained with Ponceau‐S stain and imaged with FOTO*/*Analyst PC Image software. The resulting signal was quantified and was similar between groups for all membranes. Membranes were destained in 1% TBST (50 mmol/L Tris‐HCl, pH 7.4, 150 mmol/L NaCl, 0.1% Tween 20) for 20 min, blocked in 5% milk in 1% TBST for 1 h, and then exposed to primary antibodies overnight at 4°C (Table 1). Membranes were then washed, exposed to appropriate anti‐mouse or anti‐rabbit secondary antibodies (Cell Signaling Technology, Danvers, MA) for 1 h at room temperature, washed in 1% TBST, and incubated with ECL Western Blotting Substrate for 5 min at room temperature (Thermo Fisher Scientific, Inc.). Blots were imaged on X‐ray film (Phenix Research Products, Candler, NC), and the resultant bands quantified with Carestream software. Signal from each band was normalized to mean signal from the control (thermal neutral) group.

**Table 1:** Antibodies used for western blot analysis

Antibodies	Primary dilution	Secondary dilution	Company/Product no.
Cu‐Zn superoxide dismutase (SOD1)	1:3000	1:3000	Abcam: ab16831
Mn superoxide dismutase (SOD2)	1:5000	1:3000	Abcam: ab13533
Catalase (CAT)	1:1000	1:3000	Sigma Aldrich: C0979
Cytochrome C (CYT C)	1:1000	1:3000	Cell Signaling Technology: 4280
Voltage‐dependent anion channel (VDAC)	1:500	1:1000	Cell Signaling Technology: 4661
Cytochrome c oxidase IV (COX IV)	1:500	1:1000	Cell Signaling Technology: 4850
Succinate dehydrogenase (SDHA)	1:1000	1:2000	Cell Signaling Technology: 11998
Prohibitins 1 (PHB1)	1:1000	1:2000	Cell Signaling Technology: 2426
Acetyl‐Histone H3 (Lys9)	1:1000	1:2000	Cell Signaling Technology: 9649
Peroxisome proliferator‐activated receptor gamma coactivator 1 alpha (PGC1*α*)	1:1000	1:2000	Abcam: ab72230
Estrogen‐related receptor alpha (ERR *α*)	1:1000	1:2000	Cell Signaling Technology: 13826
Sirtuin 1 (SIRT1)	1:500	1:2000	Millipore: 07‐131
Transcription factor A, mitochondrial (TFAM)	1:375	1:1000	Cell Signaling Technology: 8076
Total OXPHOS rodent WB antibody cocktail	1:1000	1:10000	Abcam: ab110413
Binding immunoglobulin protein (BIP)	1:700	1:2000	Cell Signaling Technology: 3177
Protein kinase RNA‐like endoplasmic reticulum kinase (PERK)	1:700	1:2000	Cell Signaling Technology: 5683
Inositol‐requiring enzyme 1 (IRE1*α*)	1:700	1:2000	Cell Signaling Technology: 3294

### mRNA isolation and RT‐qPCR

mRNA was isolated as recently described (Ganesan et al. 2016). Briefly, mRNA was isolated from 50 mg of powdered muscle using Direct‐zol MiniPrep column (Zymo, Irvine, CA). mRNA concentration was measured using a ND‐1000 Spectrophotometer (*λ *= 260/280 nm; NanoDrop Technologies, Inc., Wilmington, DE) and converted to cDNA via reverse transcription using a QuantiTect Reverse Transcription Kit (Qiagen, Valencia, CA). Relative transcript abundance was assessed using QuantiFast SYBR Green PCR Kit (Qiagen) for real‐time qPCR using primers in Table 2. Data were analyzed using the ΔΔCT method. All statistical analyses were performed on the ΔCT values and data are reported as fold change (2^ΔΔCT^) compared to thermal neutral.

**Table 2:** Sequences for RT‐qPCR primers

Target	Forward primer	Reverse primer
18S	AAACGGCTACCACATCCAAG	TCGCGGAAGGATTTAAAGTG
CAT	GGGTCATCTGAAAGACGCAC	CAACTTGGTGCAGGATCAGG
SOD1	ATGGTGGGCCAAAGGATCA	GATGTACACAGTGGCCACAC
SOD2	TCTGAATGCGGCTTGTTCAG	CCTAGAGGCCCCAACAGTAG

### Assessment of redox balance

Malondialdehyde (MDA) is a product of lipid peroxidation. The thiobarbituric acid‐reactive substances (TBARS) assay was used to measure MDA‐TBA adduct formation using the Cayman TBARS assay kit (cat. no 700870). The MDA‐TBA adduct was measured colorimetrically at 530–540 nm using a microplate reader (BioTek, Winooski, VT). The MDA concentration is expressed in *μ*M.

Protein carbonyl content was used as a marker of protein oxidation, which was measured using Cayman's protein carbonyl colorimetric assay kit (cat. no 10005020). 2,4‐Dinitrophenylhydrazine (DNPH) reacts with protein carbonyls and forms Schiff bases to produce hydrazone, which can be analyzed spectrophotometrically at an absorbance of 370 nm. Protein carbonyl content is expressed in nmol/mg of protein.

Catalase activity was measured according to the manufacturer's instructions (Catalase Assay Kit, Cayman Chemical Company, Item No. 707002). Measurement of catalase activity is based on its peroxidatic activity that involves alcohol of a low molecular weight as an electron donor and aliphatic alcohol as a substrate. The peroxidatic activity of catalase is determined using a reaction that converts methanol as an electron donor into formaldehyde in the presence of hydrogen peroxide. 4‐Amino‐3‐hydrazino‐5‐mercapto‐1,2,4‐trizazole (Purpald) chromogen is added to oxidized formaldehyde to allow spectrophotometric analysis. Catalase activity is expressed as nmol/min/mL/mg of protein.

Total SOD activity was measured using a commercially available kit (Superoxide Dismutase Assay Kit, Cayman Chemical Company, Item No. 706002) according to the manufacturer's instructions. SOD catalyzes dismutation of the superoxide radicals to molecular oxygen and hydrogen peroxide. SOD activity was measured using the percentage of xanthine oxidase‐generated superoxide radicals that undergo dismutation. This process is detected spectrophotometrically by a tetrazolium salt that is converted into a formazan dye in the presence of superoxide radicals. Total SOD activity is expressed as U/mL/mg of protein.

### Statistical analysis

All data were analyzed using PROC MIXED procedure of SAS version 9.2 (SAS Institute, Inc., Cary, NC). The model included time (0, 2, 4, and 6 h of heat stress) as a fixed effect. Additionally, the linear and quadratic effects of heat stress were analyzed using contrast statements of SAS. Data are reported as least square means ± SEM and considered significant at *P* < 0.05.

## Results

### Phenotypic response

The physiologic response to 2, 4, and 6 h of heat stress (HS) has been previously reported (Pearce et al. 2014). Briefly, our heating intervention caused an approximate 2°C increase in rectal temperature (39.2 ± 0.1°C to 41.2 ± 0.1°C) by 2 h compared to pigs kept at thermal neutral conditions, and pigs remained similarly hyperthermic following 4 and 6 h of heat stress (*P* < 0.05). Hyperthermic exposure caused respiratory rate to increase from 46 ± 2 bpm in the thermal neutral group to 155 ± 16 bpm, 151 ± 10 bpm, and 135 ± 6 bpm following 2, 4, and 6 h of heat stress, respectively (*P* < 0.05).

### Oxidative stress

**Figure 1:**
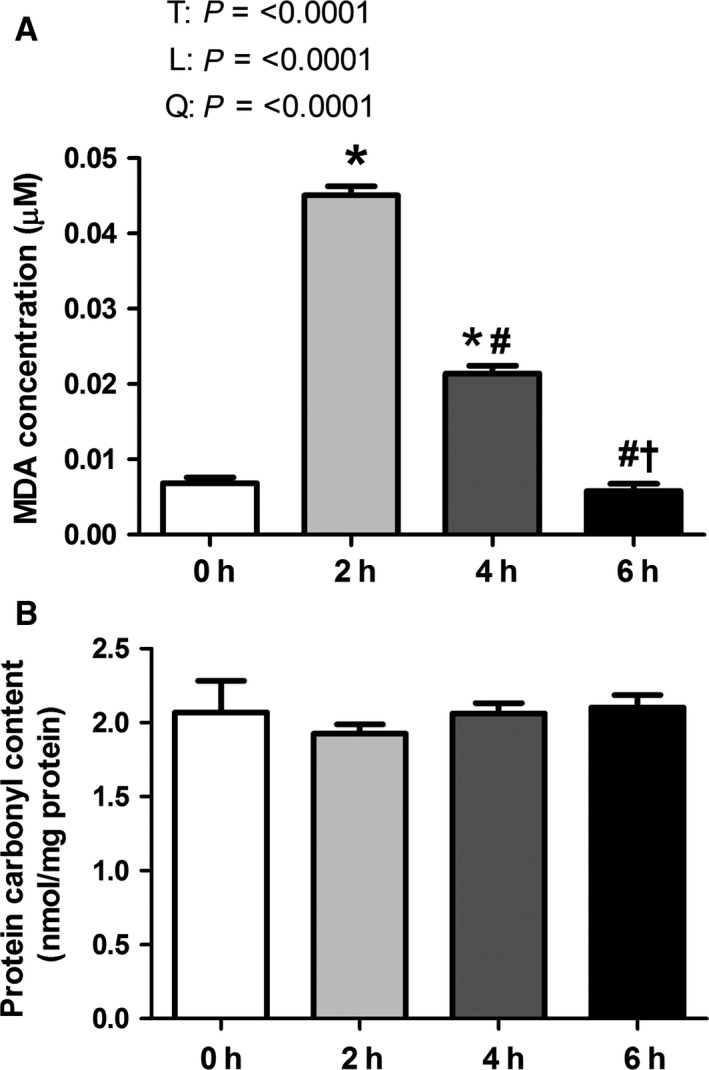
Short‐term environmental hyperthermia increased oxidative stress markers in oxidative skeletal muscle. Following 0, 2, 4, and 6 h of environmental hyperthermia, malondialdehyde (MDA) (A) and protein carbonyl content (B) were measured (*n* = 8/group). MDA concentration is expressed in *μ*M. Protein carbonyl content is expressed in nmol/mg. * indicates significant difference compared to thermal neutral control, *P* < 0.05; # indicates significant difference compared to 2 h of heat stress, *P* < 0.05; † indicates significant difference compared to 4 h of heat stress; T indicates time effect between the treatment, *P* < 0.05; L indicates linear effect between the treatment, *P* < 0.05; Q indicates quadratic effect between the treatment, *P* < 0.05.

To address our hypothesis that acute heat stress resulted in increased oxidative stress, we measured MDA concentration, a marker of lipid peroxidation. Heat stress caused a quadratic and linear effect in MDA concentration due largely to a dramatic sevenfold increase (*P* < 0.05) in MDA concentration at 2 h followed by a progressive, linear decrease following 4 and 6 h of heat stress (Fig. 1A). Heat stress increased (*P* < 0.05) MDA concentration following 2 and 4 h of heat stress compared to thermal neutral animals and animals subjected to heat stress for 6 h. Protein carbonyl content, a marker of protein oxidation was similar between groups (Fig. 1B).

**Figure 2:**
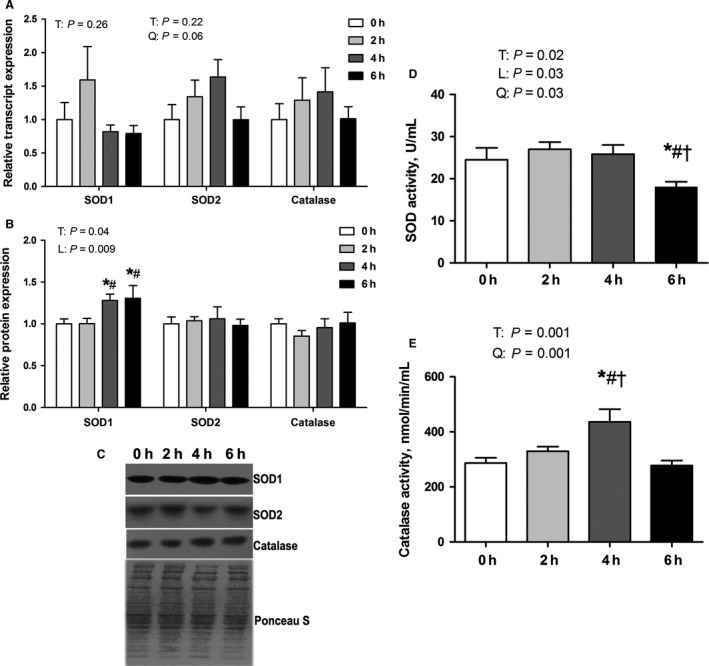
Short‐term environmental hyperthermia altered markers of antioxidant enzymes in porcine oxidative skeletal muscle. Following 0, 2, 4, and 6 h of environmental hyperthermia, transcript expression (A), relative protein abundance (B, C), and enzymatic activities (D, E) were measured (*n* = 8/group). * indicates significant difference compared to thermal neutral control, *P* < 0.05; # indicates significant difference compared to 2 h of heat stress, *P* < 0.05; † indicates significant difference compared to 4 h of heat stress, *P* < 0.05; T indicates time effect between the treatment *P* < 0.05; L indicates linear effect between the treatment *P* < 0.05; Q indicates quadratic effect between the treatment *P* < 0.05.

To determine the extent to which antioxidant enzymes may be preserving or restoring redox balance in the face of heat stress, we measured relative transcript expression of *SOD1*,* SOD2*, and *CAT*, which were similar between environmental groups (Fig. 2A). However, SOD2 transcript expression had trend toward quadratic effect (*P* = 0.06) on treatment. Relative protein abundance of SOD2 and CAT were also similar between environmental groups (Fig. 2B, C). SOD1 protein abundance was increased (*P* < 0.05) following 4 h and 6 h compared to thermal neutral and 2 h heat stress. In addition, SOD1 protein abundance increased linearly with treatment (*P* < 0.05). Because abundance is not necessarily reflective of activity, we also assessed the activities of SOD (Fig. 2D) and catalase (Fig. 2E). We found that SOD activity following 2 and 4 h of heat stress was similar to thermal neutral, however, SOD activity was decreased by 27% following 6 h of heat stress compared to other groups (*P* < 0.05). This change was preceded by a 50% increase in catalase activity following 4 h compared to thermal neutral and animals heat stressed for 2 and 6 h (*P* < 0.05). In addition, catalase activity showed quadratic effect on treatment (Fig. 2E).

### Markers of mitochondrial biogenesis and content

**Figure 3:**
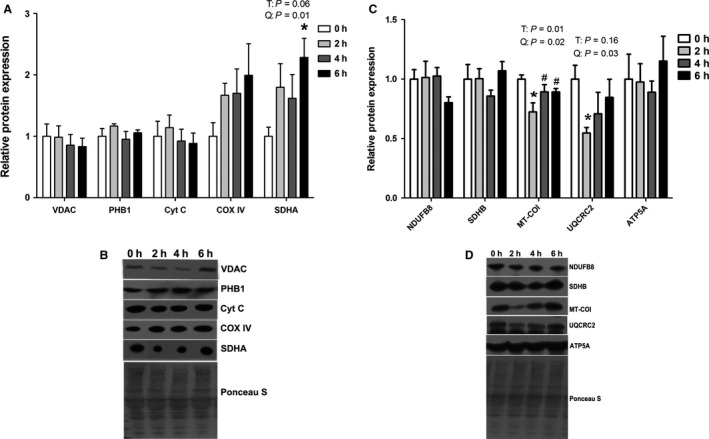
The effects of short‐term environmental hyperthermia on markers of mitochondrial abundance in oxidative skeletal muscle. Following 0, 2, 4, and 6 h of environmental hyperthermia, mitochondrial content (A, B) and oxidative phosphorylation markers (C, D) were measured using western blot (*n* = 8/group). Representative blots are included. * indicates significant difference compared to thermal neutral control, *P* < 0.05; T indicates time effect between the treatment, *P* < 0.05; Q indicates quadratic effect between the treatment, *P* < 0.05.

Given our unexpected findings in redox balance, we reasoned that decreased MDA concentration at 6 h heat stress could be explained, in part, by decreased mitochondrial content. To approximate relative mitochondrial content, abundance of several mitochondrial membrane proteins was measured as well as proteins involved in the ETC and the TCA cycle. VDAC and prohibitin found in the outer and inner mitochondrial membrane, respectively, were similar between groups (Fig. 3A, B). Likewise, cytochrome C and Cox IV, components of the ETC, were similar between groups. Relative abundance of SDHA, located on the inner mitochondrial membrane, was increased by 128% (*P* = 0.06) following 6 h of heat stress compared to thermal neutral (Fig. 3A, B). In addition, relative protein abundance of electron transport chain components including NADH dehydrogenase (ubiquinone) 1 beta subcomplex subunit 8, mitochondrial (NDUFB8; complex I), succinate dehydrogenase, subunit B (SDHB; complex II), and ATP synthase subunit *α* (ATP5A; complex V) were similar between groups (Fig. 3C, D). Protein abundance of MT‐CO1 (complex III) and UQCRC2 (complex IV) were decreased by 28% and 46%, respectively, following 2 h of heat stress compared to thermal neutral (*P* < 0.05), but was otherwise similar between groups (*P* < 0.05) (Fig. 3C, D).

**Figure 4:**
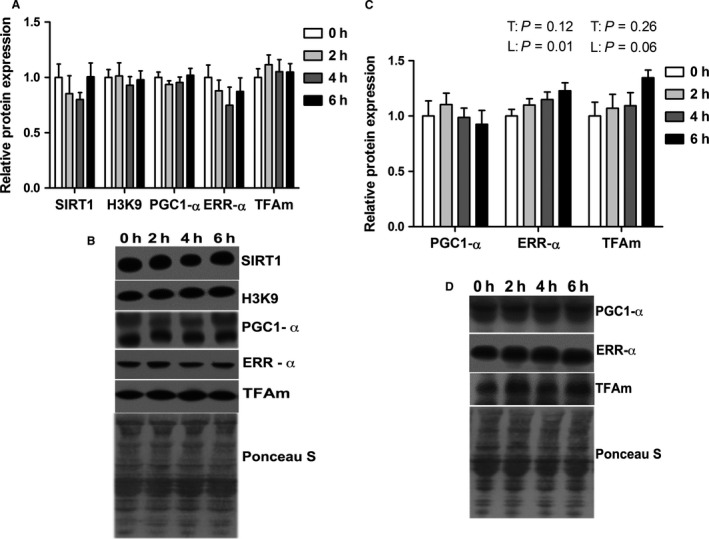
Short‐term environmental hyperthermia did not alter markers of mitochondrial biogenesis in oxidative skeletal muscle. Following 0, 2, 4, and 6 h of environmental hyperthermia, the relative protein abundance of PGC‐1*α* pathway components was measured in whole homogenate (A, B) and in nuclear fraction (C, D) by western blot (*n* = 8/group). Representative blots are included. T indicates time effect between the treatment, *P* < 0.05; L indicates linear effect between the treatment, *P* < 0.05.

To determine the extent to which mitochondrial content was maintained by biogenic signaling, we assessed activation of the PGC‐1*α* pathway as it has been established to be a master regulator of mitochondrial production. We assessed relative protein abundance of the upstream PGC‐1*α*‐activator SIRT‐1 and found that it was similar between groups (Fig. 4A, B) as was SIRT‐1 activity, assessed by measuring acetylated histone H3 lysine 9 (H3K9). Accordingly, PGC‐1*α* and downstream pathway components, ERR‐*α* and TFAm, were also similar between groups. We also measured relative abundance of PGC‐1*α*, ERR‐*α*, and TFAm in the nuclear fraction (Fig. 4C, D) were similar between groups. In addition, ERR‐*α* protein abundance (*P* < 0.05) and TFAm (*P* = 0.06) showed linear effect on treatment (Fig. 3G, H).

### Acute heat stress does not lead to endoplasmic reticulum stress

**Figure 5:**
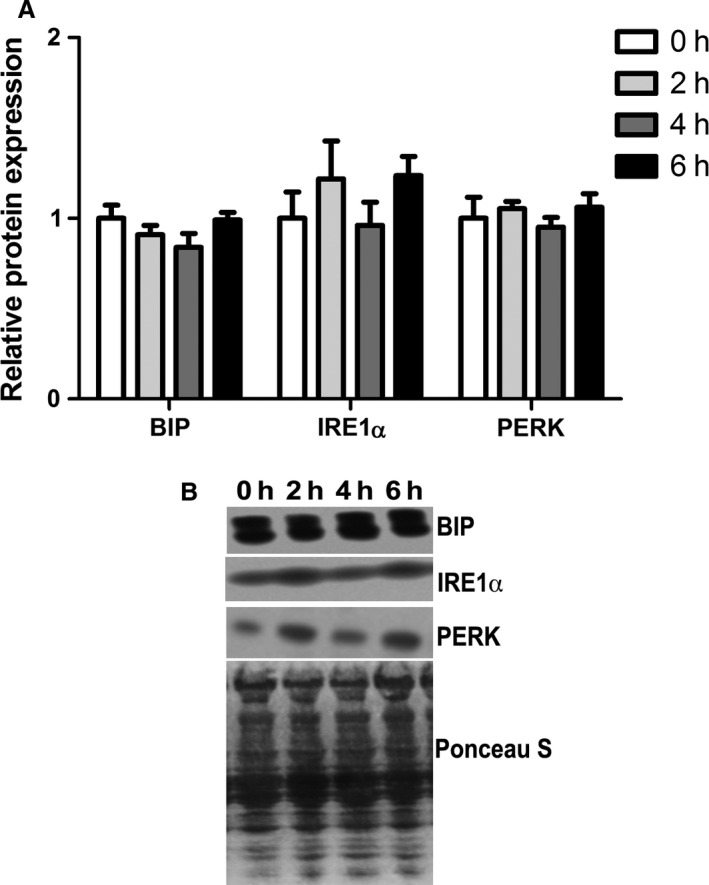
Short‐term environmental hyperthermia did not alter endoplasmic reticulum stress response markers in oxidative skeletal muscle. Following 0, 2, 4, and 6 h of environmental hyperthermia, the relative abundance of endoplasmic reticulum stress proteins (A, B) was measured by western blot (*n* = 8/group).

Given that lipid peroxidation was increased by early environmental hyperthermic exposure, we hypothesized that this would be sufficient to induce endoplasmic reticulum (ER) stress. Relative abundance of BIP, PERK, and IRE1*α* protein were similar between groups (Fig. 5A, B), indicating that short‐term environmental hyperthermia does not cause ER stress.

## Discussion

The current knowledge regarding prolonged exposure to elevated environmental temperatures clearly demonstrates its negative effects on human and animal health, animal well‐being, and agricultural economics (St‐Pierre et al. 2003). Treatment for heat stress includes general cooling and rehydration (Bouchama and Knochel 2002), but largely ignores the underlying etiology. This symptomatic approach is, in part, due to a failure to appreciate intracellular changes that occur during heat stress in a tissue‐specific manner. The current study addressed the hypothesis that short‐term heat stress results in increased oxidative stress, decreased mitochondrial content, and increased mitochondrial biogenic signaling in oxidative skeletal muscle compared to muscle from pigs maintained under thermal neutral conditions. In partial support of our hypothesis, heat stress caused a rapid but transient increase in lipid peroxidation, but not protein carbonyls. This appeared to be independent of changes in markers of mitochondrial content or biogenic signaling.

We recently found free radical injury in oxidative porcine skeletal muscle following 12 (Ganesan et al. 2017) and 24 h (Montilla et al. 2014) of heat stress but not in glycolytic skeletal muscle. Consistent with our hypothesis, we found a dramatic, though transient, elevation in lipid peroxidation (MDA) that returned to baseline values following 6 h of heat stress, while protein oxidation was similar between groups. It seems likely that lipids are more sensitive to oxidation than proteins, which was supported by previous studies showing increased plasma MDA (TBARS) concentration in human and mouse liver by supplementation of eicosapentaenoic and docosahexaenoic acids, without changes in protein carbonyls (Yasuda et al. 1998; Wander and Du 2000). We speculate that restoration or maintenance of redox balance may be in part explained by increased catalase activity following 4 h of heat stress. The subsequent reduction in SOD activity at 6 h is puzzling, though speculatively may be due to decreased mitochondrial flux driven by oxidative stress and a subsequent reduction in superoxide production. Interestingly, changes in antioxidant enzyme activity in this investigation did not result from heat stress‐modified protein or transcript expression. In contrast, increased catalase and SOD activities following 24 h of environmental hyperthermia were due, in part, to increased transcript and/or protein abundance (Montilla et al. 2014). Such findings of increased antioxidant activity with prolonged hyperthermic exposure are consistent with previous findings in porcine skeletal muscle following 3 weeks of heat stress (Yang et al. 2014). In avian muscle oxidative stress is still present following 1.5 months of heat stress as indicated by increased MDA coupled with increased SOD activity (Ghazi Harsini et al. 2012).

As lipid peroxidation may be regulated by mitochondrial respiratory substrates (Bindoli 1988), we reasoned that in addition to altered antioxidant enzyme activity, a gradual decrease in MDA concentration could be caused by decreased mitochondrial content. On the whole, markers of mitochondrial content including membrane proteins (PHB1, VDAC), the ETC (cytochrome C, COX IV), and oxidative phosphorylation were similar between groups. This makes it unlikely that decreased mitochondrial content is altering redox balance in STR muscle. In addition, the maintenance of mitochondrial content also appeared to be independent of biogenic signaling, though appreciate that 6 h of heat stress may increase biogenic signaling and subsequently mitochondrial content at future time points. This is in contrast to short‐term heat stress exposure modeling heat stroke during which mitochondrial biogenesis was stimulated in myotubes (Liu and Brooks 2012).

Oxidative stress has been shown to activate endoplasmic reticulum (ER) stress signals and inhibit refolding activity under heat stress conditions (Adachi et al. 2009; Liu et al. 2012). Despite transient increases in lipid peroxidation, we did not find changes in markers of ER stress following heat stress, which was counter to our hypothesis. This is most likely a consequence of the short‐term application of heat stress in our pigs, which was insufficient to stimulate an ER stress response.

In summary, short‐term heat stress caused a transient increase in lipid peroxidation that was countered by an increase in catalase activity independent of changes in markers of mitochondrial content, biogenic signaling, and ER stress. Importantly, the physiological response to environmental hyperthermia is the integrated response to multiple stressors including, but not limited to, heat, nutrient restriction, endotoxemia, and metabolic dysregulation. While ultimately the physiological consequence of environmental hyperthermia results from a multitude of contributing factors, the phenotypic and biochemical changes are caused by this combination; hence, this experimental approach allows the study of heat stress and these data contribute to our understanding of the early chronology of short‐term heat stress‐induced intracellular changes in skeletal muscle.

## Conflict of Interest

None declared.
